# Chloroplast Genome Annotation Tools: Prolegomena to the Identification of Inverted Repeats

**DOI:** 10.3390/ijms231810804

**Published:** 2022-09-16

**Authors:** Ante Turudić, Zlatko Liber, Martina Grdiša, Jernej Jakše, Filip Varga, Zlatko Šatović

**Affiliations:** 1 Centre of Excellence for Biodiversity and Molecular Plant Breeding (CoE CroP-BioDiv), Svetošimunska cesta 25, 10000 Zagreb, Croatia; 2 Faculty of Agriculture, University of Zagreb, Svetošimunska cesta 25, 10000 Zagreb, Croatia; 3 Faculty of Science, University of Zagreb, Marulićev trg 9a, 10000 Zagreb, Croatia; 4 Biotechnical Faculty, University of Ljubljana, Jamnikarjeva 101, 1000 Ljubljana, Slovenia

**Keywords:** chloroplast genome, annotation, inverted repeats, repeat identification

## Abstract

The development of next-generation sequencing technology and the increasing amount of sequencing data have brought the bioinformatic tools used in genome assembly into focus. The final step of the process is genome annotation, which works on assembled genome sequences to identify the location of genome features. In the case of organelle genomes, specialized annotation tools are used to identify organelle genes and structural features. Numerous annotation tools target chloroplast sequences. Most chloroplast DNA genomes have a quadripartite structure caused by two copies of a large inverted repeat. We investigated the strategies of six annotation tools (Chloë, Chloroplot, GeSeq, ORG.Annotate, PGA, Plann) for identifying inverted repeats and analyzed their success using publicly available complete chloroplast sequences of taxa belonging to the asterid and rosid clades. The annotation tools use two different approaches to identify inverted repeats, using existing general search tools or implementing stand-alone solutions. The chloroplast sequences studied show that there are different types of imperfections in the assembled data and that each tool performs better on some sequences than the others.

## 1. Introduction

Chloroplasts are organelles of plants and green algal cells specialized for photosynthesis. They are likely descended from freshwater cyanobacteria that enter a eukaryotic cell by endosymbiosis [1,2]. Accordingly, chloroplasts have their own genome, known as the plastome. The chloroplast genome is a valuable source of data for assessing plant evolution and also has potential for plant breeding programs to meet the needs of a growing world population for food, fiber, energy, and new medicines [3]. Chloroplast DNA is generally described as circular, but in some cases, it has been described as a multimeric circular, linear, or branched double-stranded molecule [4,5,6], typically 120 to 170 kbp long and consisting of 120 to 130 genes (e.g., [7,8]). It is mainly maternally inherited, but biparental and paternal inheritance is also possible. Maternal inheritance is the most common form of inheritance in ferns [9], horsetails [10], most angiosperms, cycads, and gnetophytes [11]. On the other hand, paternal inheritance has been observed in conifers [12], while biparental inheritance has been noted in the eudicot families Geraniaceae [13], Campanulaceae, and Fabaceae [14].

Four specific regions can be well distinguished in the chloroplast genome maps. The large (LSC) and small single copies (SSC) contain only single gene copies and are separated by inverted repeats (IRa and IRb) that contain completely identical genes, but in opposite or reversed orientations [15]. In most angiosperms, IRs are 20 to 30 kb long, but there are many lineages of seed and nonseed plants that have shorter or longer IRs [15,16,17]. In some extreme cases, IRs as short as 114 bp (Cryptomeria [18]) or as long as 76 kb (Pelargonium [19]) have been reported. Sequencing of the chloroplast genome of Amborella (Amborellaceae [20]) has confirmed the presence of IRs in basal angiosperms. Thus, the presence of IRs in the chloroplast genome may be an ancient trait that was probably already present in the plastome of the common ancestor of flowering plants.

The presence of IR regions in the chloroplast genome is of functional significance. The presence of duplicated rRNA genes in chloroplast genomes could be a selective advantage that allows higher production of proteins in a short time. They also play a role in replication initiation [21], genome stabilization [22], and gene conservation [23]. Intramolecular recombination between IR copies, known as flip-flop recombination, has been proposed as a mechanism that prevents divergence and has the potential to reverse the polarity of chloroplast DNA segments [15,24,25]. In addition to the absence of IR regions in the chloroplast genome, their massive expansion or reduction in size could also have a negative impact on the structural stability and uniformity of the plastome [26,27,28,29,30,31]. Loss of one copy of the inverted repeat has been reported in legumes [32,33,34], conifers [35,36,37], members of the family Geraniaceae [38,39], Cactaceae [40], Arecaceae [41], Passifloraceae [42], and most nonphotosynthetic and parasitic plants in the Orobanchaceae family [3,11,43,44].

Due to numerous advantages (simple structure, mostly uniparental mode of inheritance, haploidy, slow evolutionary rate, etc.) compared to nuclear genomes, chloroplast DNA data have been used extensively in studies of plant molecular evolution, phylogenetics, and phylogeography [45]. Advances in DNA sequencing have increased the affordability of genome data, and furthermore, the small size of the genome and the large number of cpDNA molecules [21,46] have made the assembly of complete chloroplast sequences much easier than the assembly of complete nuclear genome sequences [47]. This is also reflected in a large number of available cpDNA sequences in public databases [48]. At the time of writing, more than 8000 plant cpDNA sequences were available in the National Center for Biotechnology Information (NCBI) database, as opposed to the number of available plant nuclear sequences, which is at least an order of magnitude smaller [49].

A large amount of public cpDNA data raised questions about the format consistency of chloroplast data, such as the order and orientation of cpDNA structural parts [50] and the different formats for storing the locations of IRs [51]. The most important steps in genome projects are sequencing, assembly, and annotation of the sequences [52]. Sequencing can be performed using a variety of technologies [53]. The assembly of cpDNA sequences can be performed using standard de novo assemblers, e.g., SOAPdenovo [54] and ABySS [55], or specialized organelle or plastome assemblers, e.g., Fast-Plast [56] and GetOrganelle [57]. Due to the peculiarities of the chloroplast genome, cpDNA sequences are annotated using specialized tools, e.g., DOGMA [58] and GeSeq [59]. These tools aim to annotate the expected cpDNA genes and the locations of inverted repeats.

From a bioinformatic perspective, the problem of identifying IRs can be formulated quite simply: in a circular genome, identify the two longest regions that are exact reverse complements. This problem is easily solved with a broad range of standard software programs (e.g., Blast [60] or REPuter [61]) designed to identify repeats. Developing a solution to this problem and confronting it with practical requirements broadens the range of issues to be considered. The main problem is imperfections that can be introduced into genome sequences at earlier steps through sequencing and/or assembly. If we expect imperfections, we cannot expect the IRs to be identical complementary copies, and we must tolerate some differences. Then, we get to the issue of what kind of mismatches we can tolerate and, if possible, why. DNA sequencing produces a certain percentage of erroneous base calls [62] that can potentially affect the final assembly result. The errors caused by sequencing are likely to be very short substitutions of indels.

The assembly itself is a complicated problem, and it is not possible to implement a method that theoretically finds the perfect solution [63]. All methods use heuristics to make the implementation feasible and produce solid results, but by making it possible, some questionable situations can lead to an assembly that is far from perfect. This type of error is very difficult to detect, and it is even difficult to describe what type of error to expect. It is likely that both short and long substitutions and indels will occur during assembly. Two other points concerning implementation are as follows: (a) the chloroplast genome is usually circular, but the sequence is represented in linear form, and (b) there may be ambiguous base pairs in the sequence. If oversight is made in the implementation, the results may be of lower quality. In most cases, such implementation errors can be easily detected and corrected. Although studies have been performed on the quality and consistency of gene annotation of several cpDNA annotation tools [64,65], we are not aware of any studies that have analyzed and compared the methods used to identify IRs.

The aim of this study was to analyze the methods used to identify IRs in six existing cpDNA annotation tools. The tools analyzed were Chloë [66], Chloroplot [67], GeSeq [59], ORG.Annotate (https://git.metabarcoding.org/org-asm/org-annotate, accessed 31 December 2021), PGA [68], and Plann [69]. We investigated how annotation tools overcome problems in identifying IRs. The methods were analyzed in two ways: through qualitative analysis of the program code and the articles in which it is described and through quantitative analysis of the results obtained with these methods. We tested the results on more than 4000 complete chloroplast genomes from the asterid and rosid clades.

## 2. Results

### 2.1. Comparative Code Analysis

For each annotation tool, we extracted the description of the IR identification strategy from the respective publications and analyzed the source code to find the exact strategy that was implemented. In general, bioinformatic solutions for identifying IR can be implemented by using existing general search tools or as stand-alone solutions. Table 1 provides descriptions of the IR identification strategies described in the original publication and what we deduced from the code analysis.

Three tools (Chloë, Chloroplot, GeSeq) implement stand-alone solutions. The strategies of Chloë and Chloroplot are very similar. They merge small blocks (up to 100 bp) of exact inverted repeats into larger regions and tolerate small differences. GeSeq implements a different strategy by finding relatively large blocks (at least 2000 bp) of identical inverted repeats that define the IR ends and considers the regions in between as IRs. Chloë and Chloroplot focus on the circularity of the chloroplast genome resulting from code analysis. In the case of GeSeq, it is not possible to deduce how it handles genome circularity. As mentioned earlier, GeSeq uses the OGDRAW tool to identify IR. However, OGDRAW does not handle circularity by itself. With appropriate formatting of the input data (e.g., the input of a duplicated cpDNA genome) or with additional processing of the OGDRAW results (e.g., expansion of the regions found), it is possible to cover genome circularity, but we do not know the exact application because no code is available for GeSeq.

The method implemented in Chloë identifies IRs with three numbers: start position of IRa, start position of IRb, and IR length. Thus, it is not possible for the IRa and IRb annotated by Chloë to differ in length. The results of the GeSeq method depend strongly on the input data. It can allow very large differences in the inner region of IRs, but it can also truncate IRs found, even with a single base difference or a single ambiguous character. From the GeSeq source code, it appears that end blocks of at least 2000 base pairs in length must not contain ambiguous characters.

The original publication describing GeSeq [59] states that the strategy is to identify identical IR pairs, while the method used tolerates mismatches. This is because GeSeq now uses the OGDRAW method for IR identification.

The strategy implemented in ORG.Annotate involves detecting approximate IR positions and then refining the results based on querying a database included in the package, which contains a set of 45 LSC and 72 SSC regions. The implementation uses repseek [70] and blast [60] for IR identification and to process the results of both methods. Both repseek and blastn queries are performed on the original cpDNA genome (i.e., not on the duplicated genome as in PGA) and therefore ORG. Annotate ignores the circularity of the genome.

Two tools (PGA, Plann) use the blastn program to identify repeats in a complete chloroplast sequence via a self-blastn search. Both programs use very similar arguments, except that PGA implements the search for repeats in the duplicated genome, while Plann restricts the search to the original genome. Therefore, similarly to ORG.Annotate, Plann ignores genome circularity.

Regarding the flexibility of programs in tolerating sequence differences between IRa and IRb, methods using existing software to identify IR (ORG.Annotate, PGA, Plann) are expected to allow differences in a consistent manner, as this depends on the argument values passed to specialized tools (blastn, repseek). For the other methods, it is difficult to determine exactly how much difference they tolerate, as this is neither stated in the documentation nor clear from the code.

Each method has a theoretical minimum length of the putative IRs. For Chloë, Chloroplot, and GeSeq, the minimum length is equal to the length of the block size used, i.e., 100, 1000, and 2000 bp, respectively. ORG.Annotate does not filter repeats by size, so the minimum length is a few dozen bp, which corresponds to the shortest blastn output. When searching for IRs, PGA and Plann exclude repeats smaller than 1000 and 10,000 bp, respectively.

### 2.2. Comparative Results Analysis: Overview

**Figure 1:**
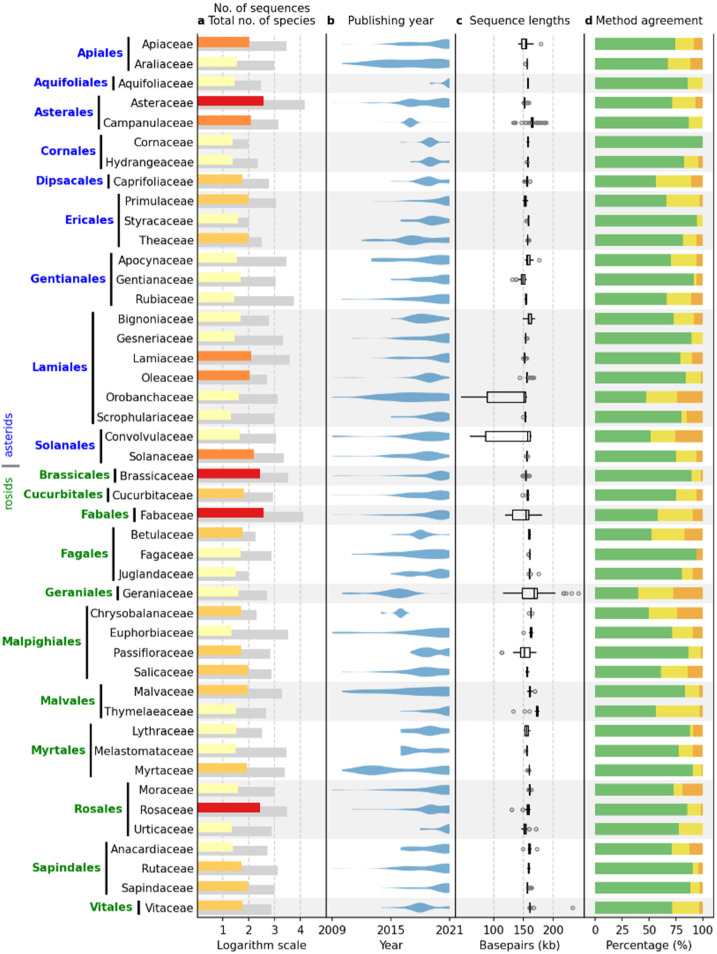
Availability of the complete chloroplast genome, publishing years, sequence lengths, and the outcome of IR identification using six annotation tools in different families of asterids and rosids. Represented families contain 20 or more sequences. (**a**) Total number of species (gray bar) and number of available complete chloroplast sequences (colored bar) in NCBI, where yellow represents 20–49, orange 50–99, red 100–199, and brown ≥200 sequences. (**b**) Violin plots of the number of sequences published in NCBI per year. (**c**) Box plots of complete chloroplast sequence lengths. (**d**) The proportion of different outcomes of IR identification: six annotation tools produced the same outcome (green), two different outcomes (yellow), and three or more different outcomes (orange). Outcomes were treated as the same if the lengths of the identified IR differed by less than 10 bp.

Figure 1 shows plant families with 20 or more sequences, including data from both the IRL and IR datasets. The data presented include 3623 sequences, representing 87.6% of the total dataset. The asterid and rosid clades are represented by 1688 and 1935 sequences, respectively. The largest families per clade are Asteraceae (asterids) and Fabaceae (rosids), with 382 and 384 sequences, respectively. The total number of species per family included in the NCBI Taxonomy Database (https://www.ncbi.nlm.nih.gov/taxonomy, accessed on 31 December 2021) ranges from 95 (Cornaceae) to 14,245 (Asteraceae) in the asterid clade and from 99 (Juglandaceae) to 12,782 (Fabaceae) in the rosid clade. In total, 4.32% of the species in the dataset had assembled cpDNA sequences.

**Figure 2:**
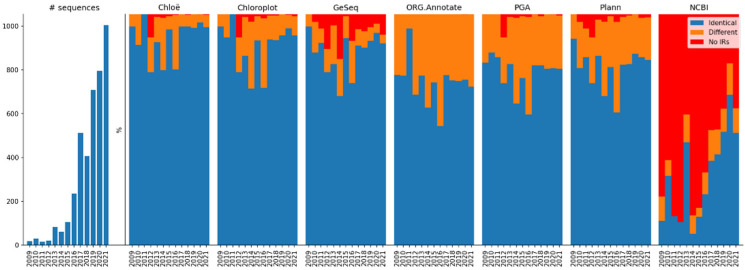
Number of complete chloroplast sequences added to GenBank per year (leftmost chart) and the relative ratio of the three types of sequences identified using six annotation tools (and those annotated in NCBI): blue—identical IRs, orange—different IRs, and red—no IRs.

As expected, most complete chloroplast sequences have been published recently, with more than 85% of all sequences published in the last five years (Figure 2). Continuous sequencing efforts were observed in most families (Figure 1b), especially in the families Asteraceae (asterids, 382 sequences) and Fabaceae (rosids, 384 sequences), with some exceptions where almost all sequences were published within a short period of time. Examples are the families Campanulaceae (asterids, 121 sequences) and Chrysobalanaceae (rosids, 50 sequences), where most sequences were produced in 2016 and 2017, respectively, probably as a result of a single, rather isolated research effort.

The median of the complete chloroplast sequence length ranged from 149,100.5 (Gentianaceae) to 165,063 (Campanulaceae) in the asterids and from 151,362 (Passifloraceae) to 173,148 (Thymelaeaceae) in the rosid clade (Figure 1c). In general, the interquartile range (IQR) length within a family was low, ranging from 59.5 (Vitaceae, rosids) to 8463 (Apiaceae, asterids). However, five families were clear outliers with IQRs of 14,852 (Passifloraceae, rosids), 24,753.5 (Geraniaceae, rosids), 26,909.5 (Fabaceae, rosids), 64,236.5 (Orobanchaceae, asterids), and 75,590.5 (Convolvulaceae, asterids).

Identical results of all six annotation tools were observed in 75.16% of the sequences, while two and three or more different results were obtained in 17.97% and 6.87% of the sequences, respectively (Figure 1d). Identical results per family dataset ranged from 40% to 100% of the sequences, with the lowest agreement among the six annotation tools observed in the Geranicaceae (rosids).

### 2.3. Comparative Results Analysis: IRL Dataset

The number of sequences in the IRL dataset in which IRs were identified is shown in Table 2, while detailed identification results can be found in Table S1.

ORG.Annotate identified IRs in almost all sequences, Chloroplot identified IRs in 32% of sequences, while the other methods identified IRs in 20% or fewer sequences. In most cases, however, the regions identified as IRs were between a few hundred and a few thousand bases long. The number of sequences in which the regions of at least 10,000 bp in length ranged from four to eleven, depending on the method (Table 2). As expected, no sequence containing IRs longer than 10,000 bp was identified in most families included in the IRL dataset. The exceptions were the family Ericaceae (asterids) and the genus *Erodium* (Geraniaceae, rosids).

The family Ericaceae was represented by 16 complete chloroplast sequences of species belonging to nine genera. IRs were identified in eight of 16 sequences using three annotation tools (Chloë, ORG.Annotate, Plann). The genera without identified IRs were *Allotropa* (*A. virgata* Torr. & A.Gray), *Hemitomes* (*H. congestum* A.Gray), *Monotropa* (*M. hypopitys* L., *M. uniflora* L.), *Monotropsis* (*M. odorata* Schwein. ex Elliott), and *Pityopus* (*P. californicus* (Eastw.) Copel.). In the genus *Rhododendron*, four of six sequences contained IRs. These four species were *R. delavayi* Franch. and *R. kawakamii* Hayata with IRs 24 kb in length, and *R. griersonianum* Balf.f. & Forrest and *R. platypodum* Diels with IRs 47 kb in length. *Rhododendron datiandingense* Z.J.Feng and *R. simsii* Planch. did not contain IRs. Genera in which all available sequences contained IRs were *Agapetes* (*A. malipoensis* S.H.Huang, 32 kb), *Gaultheria* (*G. griffithiana* Wight, 32 kb), and *Vaccinium* (*V. macrocarpon* Aiton, 34 kb; *V. oldhamii* Miq., 31 kb).

The genus *Erodium* was reported to lack IRs because of evidence of loss of IR in *E. carvifolium* Boiss. & Reut. [38] and *E. texanum* A.Gray [39], for which complete chloroplast sequences were available at that time. We confirmed that IRs were missing in an additional five species (*E. crassifolium* L’Her., *E. manescavi* Coss., *E. reichardii* (Murray) DC., *E. rupestre* (Pourr.) Guitt., *E. trifolium* (Cav.) Guitt.). Nevertheless, five annotation tools (Chloë, Chloroplot, GeSeq, PGA, Plann) identified IRs in three other species (*E. absinthoides* Willd., 45 kb; *E. chrysanthum* L’Her., 47 kb; *E. gruinum* (L.) L’Her., 25 kb) of the genus.

### 2.4. Comparative Results Analysis: IR Dataset

The IR dataset, containing 3996 complete chloroplast sequences, was processed to analyze the following properties of the six annotation tools: (a) type of IR regions identified, (b) treatment of circularity of the chloroplast genome, (c) treatment of sequences with ambiguous bases, and (d) differences between IRa and IRb. Finally, we analyzed the length distribution of the identified regions at different taxonomic levels. The detailed identification results for the IR dataset can be found in Table S2.

#### 2.4.1. Type of IR Regions Identified

After processing 3996 sequences of the IR dataset using six annotation tools (and those annotated in NCBI), we classified them into three types: (a) identical IRs, (b) different IRs, and (c) no IRs, as explained in the Section 4. The IR identification results in the IR dataset are shown in Table 3.

The Chloë and Chloroplot methods found a large number of identical IRs and a relatively small number of different IRs, which is related to their strategies tolerating only small differences. The result of the GeSeq method was similar, but this is due to the property of the method that IRs must have ends that match by at least 2000 bp. The ORG.Annotate method annotated almost all sequences with 69.6% identical IRs and 30.3% different IRs. This is because the tool follows the most liberal strategy for tolerating differences. The PGA and Plann methods found a relatively small number of identical IRs and a large number of different IRs, which is related to their similar use of blastn.

It is expected that the recently added complete chloroplast sequences will be of better quality than the older sequences because sequencing technologies and assembly software have rapidly improved. Figure 2 shows the relative ratio of sequences with identified IRs (identical and different) and without IRs per year of sequence publication. Nevertheless, the performance of all annotation tools was consistent, and newer sequences were annotated at a similar ratio to older ones. This is especially true for the last five years, when more than 85% of the sequences in the entire dataset were added. However, a large proportion of the complete chloroplast sequences stored in NCBI does not yet have annotated IRs (43.37%; Table 3).

#### 2.4.2. Treatment of Circularity of the Chloroplast Genome

Another bioinformatic problem for the annotation tools is how to deal with the circularity of the chloroplast genome, since the complete chloroplast sequences are represented in linear form. Therefore, in some cases, the sequence must be wrapped to identify IRs. To check how the different annotation tools handle circularity, we counted how many cases identified IRs at the first attempt (without wrapping) and with wrapping (Table 4).

The methods implemented in ORG.Annotate and Plann did not find additional sequences with IRs because these tools cannot handle the circularity of the chloroplast genome, as also shown by code analysis.

Chloë, Chloroplot, and PGA performed similarly well, finding IRs in an additional 500 to 700 sequences by wrapping. GeSeq annotated significantly fewer IRs by wrapping. As mentioned earlier, it is not possible to infer the properties of GeSeq when dealing with chloroplast sequences that need to be wrapped. The results show that all four methods (Chloë, Chloroplot, GeSeq, PGA) annotated a very similar number of sequences (~30) that needed to be wrapped for more than 2000 bp (Table S2). In the case of sequences that needed to be wrapped less than 2000 bp, GeSeq identified IRs in only 41, while Chloë, Chloroplot, and PGA annotated more than 500 sequences. This outcome suggests that GeSeq does not use a duplicated cpDNA genome as an input to ODGRAW methods, but extends the found regions around the sequence origin.

#### 2.4.3. Treatment of Sequences with Ambiguous Characters

In the IR dataset, there were 526 (13.16%) complete chloroplast sequences that contained ambiguous characters (Table 5). As expected, for all methods, the proportion of sequences with different IRs or no IRs was considerably larger in the dataset containing ambiguous characters than in the dataset without ambiguous characters. All methods performed similarly well, failing to identify IRs in only 0 to 2.09% of sequences, with the exception of GeSeq. The GeSeq method did not annotate IRs in 43.2% of sequences, consistent with the results of the code analysis that IR end blocks (~2000 bp) may not contain ambiguous characters.

#### 2.4.4. Differences between IRa and IRb

All analyzed annotation tools can identify different IRs. The number of sequences with different IRs ranged from 264 (Chloë) to 1212 (ORG.Annotate) (Table 6). We analyzed differences in lengths, between IRa and IRb, in identified IRs. The annotation tools differed significantly in the number of sequences identified, where the IRs differed in length. The Chloë method did not annotate IRs with different lengths in agreement with the results of the code analysis. The remaining annotation tools can be divided into two groups. The first group, consisting of Chloroplot and GeSeq, annotated relatively few IRs of different lengths compared to the second group (ORG.Annotate, PGA, Plann). This can be explained by the fact that the methods of the first group implemented a stand-alone identification strategy, whereas the methods of the second group used specialized software to identify repeats (repseek, blastn).

#### 2.4.5. Method Agreement

Six annotation tools were compared based on the agreement in identified IRs. For a pair of methods, we counted the number of sequences where the resulting IRs of the first method were longer than those of the second method. To exclude very similar results, we assumed that one IR was longer than the other if its length differed from the other by more than 10 bp (Table 7).

Pairwise comparisons showed that the annotation tools gave the same result for 3150 to 3663 of 3996 sequences in the IR dataset. This result is consistent with the results presented in Figure 1d, which shows that the overall agreement between the methods is 75.16%.

The number of longer IRs identified ranged from 10 (0.25%) sequences in the case of Chloë/PGA to 833 (20.85%) sequences when comparing PGA and GeSeq. PGA identified the longest IRs compared to any other tool.

For all method pairs, there were sequences where using one method resulted in longer IRs than using another, suggesting that each method covers some instances of input data better than another.

#### 2.4.6. IR-Length Statistics

**Figure 3:**
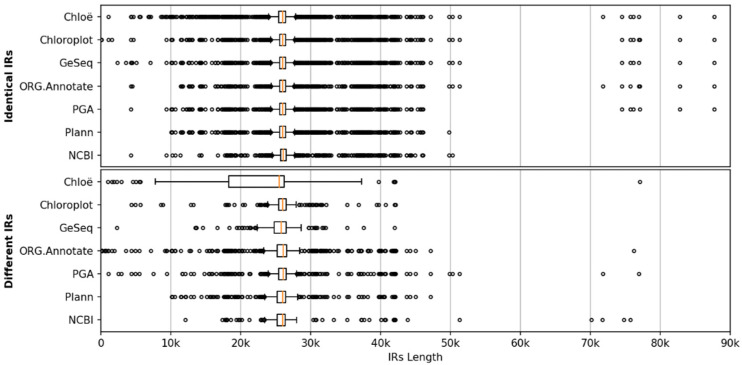
Box plots of the IR length distributions in complete chloroplast genome sequences grouped by type of IRs identified (identical or different) using six annotation tools (and those annotated in NCBI).

Box plots of the IR length distributions in complete chloroplast genome sequences grouped by type of IRs identified (identical or different) using six annotation tools are shown in Figure 3. The length distributions of the identical IRs were very similar for all methods. The medians ranged from 25,992 to 26,034 bp, and the interquartile range (IQR) was between 827.75 and 865.75 bp, with Chloë having a slightly wider IQR (968.25 bp), whereas the Plann method did not annotate any IRs longer than 50 kb, in agreement with the results of the code analysis. For the different IRs, the length distribution of Chloë differed considerably from the other methods. The median for Chloë was 25,494.5 bp, whereas for the other methods, the medians ranged from 25,807.5 to 26,050.5 bp. Similarly, the IQR for Chloë was much wider (7926.5 bp) than that of the other methods (1008.00–1678.25 bp). Using all methods, the length distribution of identical IRs was narrower than the distribution of different IRs. This was expected, since IRs that differ were more likely to be problematic, and these annotations are probably shorter than they should be in the case of perfect matches. This was most pronounced for Chloë, where the length distribution of different IRs was considerably stretched toward lower lengths.

The distributions show two distinguishable groups of sequences with longer IRs as outliers, the first with IRs longer than 70 kb and the second with lengths between 43 and 52 kb.

The sequences of the first group belong to seven of 22 species of the genus *Pelargonium* (Geraniaceae) with IRs between 75 and 88 kb (*P. dolomiticum* R.Knuth, 77 kb; *P. endlicherianum* Fenzl, 83 kb, *P. quinquelobatum* Hochst. ex Rich., 77 kb; *P. spinosum* Willd., 76 kb; *P. transvaalense* R.Knuth, 87 kb; *P. trifidum* Jacq., 75 kb; *P. x hortorum*, 76 kb), which is consistent with previous results of a highly rearranged genome and greatly expanded inverted repeat in *Pelargonium* species [19,71]. In addition, three species of the genus *Pelargonium* contained IRs of approximately 45 kb (*P. exhibens* Vorster, 45.6 kb; *P. myrrhifolium* (L.) L’Her., 45.3 kb; *P. tetragonum* (L.f.) L’Her., 45.7 kb).

Three methods identified IRs of 72 kb in the sequence of *Vitis romanetii* Rom.Caill. The genus *Vitis* contained 52 sequences in the dataset. All sequences, except *V. romanetii* and *V. yunnanensis* C.L.Li, were of very similar length (161,100 ± 300 bp) and contained uniform IRs of 26,340 ± 50 bp. The sequences of *V. romanetii* and *V. yunnanensis* were 232 and 167 kb long, containing IRs of 72 and 32 kb, respectively. Similar results were demonstrated by [72].

The second group of outliers contained sequences from the genera *Cyphia* (Campanulaceae) and *Passiflora* (Passifloraceae). The genus *Cyphia* was represented by nine sequences, seven of which contained IRs longer than 43 kb, and two contained slightly shorter IRs (40–42 kb). There were 48 sequences from the genus *Passiflora*, of which five contained IRs of 44 kb or more, while most of the other sequences had IRs between 20 and 30 kb.

## 3. Discussion

Most cpDNA genomes have a quadripartite structure. This structure is established with two copies of a large inverted repeat (IR) separating large (LSC) and small (SSC) single copy regions [15,73]. In the sequencing of the cpDNA genome, the identification of IRs is performed in the annotation step.

In general, the identification of IRs in the genome is considered a simple task, but in some cases, the results obtained may not be satisfactory. There are two reasons for this.

The first reason is related to the treatment of the biological properties of the chloroplast genome, including the circularity of the genome and the decision on the minimum and the maximum length of the inverted repeat. This treatment is not always adequately implemented in annotation tools. The second reason is related to data quality, which is very difficult to assess or even detect because there are no gold standards for genome assembly [74]. Biologically, IRs should be regions of exact inverted complements. However, it would be advisable to allow cases where small differences are found between regions rather than being too rigid. It is quite difficult to specify how many differences can be tolerated, and the same is true for ambiguous characters. From a bioinformatic point of view, these problems are usually relatively easy to find and correct once thresholds are established.

The existence of specialized chloroplast annotation tools that do not annotate IRs at all (AGORA, CpGAVAS2, and MFannot) suggest that knowledge of chloroplast structure is less important than knowledge of the genes present in cpDNA. This indicates that gene data are more widely used in subsequent research. In the IR dataset, which consists of sequences expected to contain IRs, IR annotations are missing in 43.37% of the cases in the NCBI data, including 40.74% of the sequences published in 2021. This suggests that there is room for improvement in the stored data, and that caution should be exercised when submitting a sequence to GenBank.

### 3.1. State of the Art

Chloroplast annotation tools identify IRs with different strategies. The main reason for trying different approaches to address a relatively simple bioinformatic problem is the awareness that IRs can be found that are not exact copies. In general, annotation tools allow for small differences between IRa and IRb. This is usually omitted from the articles describing the tools. The only exception is the documentation for Chloroplot, which clearly refers to the possibility of detecting nonidentical IRs and whose web application visualizes the differences found [75], but without documenting the parameters that control the process.

Classification of the identified IR regions by type (identical IRs, different IRs, no IRs) reveals two groups of annotation tools. The first group implements standalone solutions (Chloë, Chloroplot, GeSeq), while the second group uses blastn (ORG.Annotate, PGA, Plann). The annotation tools of the first group identify a higher percentage of identical IRs (~90%) than those of the second group (70–80%). This is because the tools of the first group use specific matching techniques with a limited number of possible misalignment patterns, while the tools of the second group use existing alignment solutions based on the mismatch score, which results in tolerating more different misalignment patterns.

Two (ORG.Annotate, Plann) of the six annotation tools do not treat sequences as circular, and one does so only partially (GeSeq). This suggests that implementation problems are more common than we might expect. It is also possible that at the time these tools were developed, there were few sequences to test, whereas currently, there are many complete cpDNA data to work with and test different input data.

There is a notable difference in the results of IR identification for sequences with and without ambiguous characters. All annotation tools, but especially the GeSeq method, have a lower success rate in dealing with sequences with ambiguous characters. This is to be expected, as ambiguous characters lead to uncertainties in the data, and this situation is more difficult to handle.

Among the six tools analyzed (Chloë, Chloroplot, GeSeq, ORG.Annotate, PGA, and Plann), there are six different approaches that have their own expectations about the kinds of differences that might be found. Each of these approaches addresses some types of differences, but none of them addresses all differences that can reasonably be tolerated. Considering these findings, it is not possible to state the best strategy for identifying IRs, since all strategies cover some situations with cpDNA data.

### 3.2. Further Research

Although there are many reports of taxa without inverted repeats, further research is clearly needed on the mechanism of IR loss and its taxonomic implications, as well as on the relationship between taxonomic relatedness and IR sequence length. In general, the analysis of taxa without inverted repeats confirmed all previous results [38,39,76,77,78,79,80,81,82,83,84], but two cases require additional clarification. First, IRs are absent from eight of 16 sequences in the family Ericaceae. The genera without IRs are *Allotropa* (*A. virgata* Torr. & A.Gray), *Hemitomes* (*H. congestum* A.Gray), *Monotropa* (*M. hypopitys* L., M. uniflora L.), *Monotropsis* (*M. odorata* Schwein. ex Elliott), and *Pityopus* (*P. californicus* (Eastw.) Copel.). Two other species in the *Rhododendron* genus (*R. datiangingense* Z.J.Feng, *R. smisii* Planch.) also have no IRs, while *R. delavayi* Franch., *R. kawakamii* Hayata, *R. griersonianum* Balf.f.& Forrest, and *R. platypodum* Diels have IRs. Genera in which the available sequences contain IRs are *Agapetes* (*A. malipoensis* S.H.Huang), *Gaultheria* (*G. griffithiana* Wight), and *Vaccinium* (*V. macrocarpon* Aiton and *V. oldhamii* Miq.). The second case concerns the genus *Erodium*, in which seven out of 10 sequences lack IRs. IRs are absent in *E. carvifolium* Boiss. & Reut., *E. crassifolium* L’Her., *E. manescavi* Coss., *E. reichardii* (Murray) DC., *E. rupestre* (Pourr.) Guitt., *E. trifolium* (Cav.) Guitt. and *E. texanum* A.Gray, while *E. absinthoides* Willd., *E. chrysanthum* L’Her., and *E. gruinum* (L.) L’Her. have IRs. Thus, the genera *Rhododendron* and *Erodium* could serve as model genera for studying the mechanism of IR loss.

The distributions of the lengths of the identified IRs show that, in general, all annotation tools give similar results, especially in cases where identical IRs are detected. The median length of the IRs was ~26 kb with an interquartile range (IQR) of ~800 bp. Further analysis of IR length distributions at lower taxonomic levels (i.e., within families or even genera) would be required to assess the relationship between taxonomic relatedness and the IR length of sequences.

Finally, in view of the standardization of cpDNA sequence data and the possible influence of nonstandardized data on phylogenetic analysis, it is advisable to standardize these sequences according to [50].

Although sequencing and assembling the complete chloroplast genome is a far simpler task than assembling nuclear DNA [47], the sequences obtained are not error-free. This problem could be further investigated by checking the length differences between IRa and IRb. All annotation tools are able to identify IRs of different lengths, sometimes by even more than 100 bp. Additional studies can be performed to determine the types of differences between the regions. Since IRs should be biologically identical, these differences may indicate possible errors in sequencing or assembly. Similarly, the quality of an assembly is checked by mapping to a reference genome [85]. The differences are either substitutions or indels. It is likely that short substitutions indicate sequencing errors, whereas indels and long substitutions indicate assembly errors.

If a new annotation tool is to be introduced, we think it best to adopt ideas from all existing strategies. First, self-alignment covers all cases of identical or nearly identical IRs (e.g., PGA and Plann). If IRs cannot be identified in this way, the self-aligned parts can be merged by checking the intervening sequences for allowable differences (e.g., Chloë and ORG.Annotate). If merging does not result in IRs, an inspection of the larger self-aligned fragments can find the junction points (e.g., Chloroplot and GeSeq) of the IRs, and at least indicate the possible location of the IRs. Further data analysis can be performed when developing a new method to determine what thresholds should be used for the alignment parameters and what types of differences are acceptable. Fortunately, a growing number of complete cpDNA sequences are available for the analysis and verification of possible solutions.

## 4. Materials and Methods

### 4.1. Data Acquisition

The complete chloroplast genome sequences were downloaded from NCBI (http://www.ncbi.nlm.nih.gov, accessed on 31 December 2021). We acquired all genomes for asterid and rosid clades available at this time and created two datasets. The inverted repeat-lacking (IRL) dataset contained the sequences of taxa for which the loss of IR had been documented. The remaining sequences, which we assumed to have the standard quadripartite structure, formed the IR dataset.

A total of 4137 sequences were used for the analyses, of which 1924 were from asterids and 2213 were from rosids. From the data, we created IRL and IR datasets containing 141 and 3996 sequences, respectively (Table 8). The list of sequences included in the IRL dataset with their taxonomic information and references is given in Table 9. The genus *Monsonia* (Geraniaceae, rosids) was included in the IRL dataset because [82] found no IR in *M. emarginata* (L.f.) L’Her. or *M. marlothii* (Engl.) F.Albers. Although [39] identified IR in *M. speciosa* L., it was reduced to only 7 kb.

On the other hand, the genus *Passiflora* was included in the IR set, although [42] reported that two of 46 species of the genus lacked IR, namely, *P. capsularis* L. and *P. costaricensis* Killip.

### 4.2. Comparative Code Analysis

We examined chloroplast annotation tools that were available at the time of writing. Other tools implemented as web applications, such as CGAP [86], CpGAVAS [87], and Verdant [88], are no longer functional (as of 23 November 2021). In addition, the software DOGMA [58], which has been widely used over the past decade, is “being sunsetted after 15 years and will not be available for use in the near future,” according to its website (https://dogma.ccbb.utexas.edu/). Some tools are limited to gene annotation and do not annotate inverted repeats (accessed on 23 November 2021), such as AGORA [89], CpGAVAS2 [90], and MFannot [91]. Note that in [87] on CpGAVAS, the predecessor of CpGAVAS2, it is stated that “the inverted repeats are identified using the vmatch software tool with default parameters” while in [90] on CpGAVAS2, there is no mention of the IR identification strategy implemented.

To investigate how annotation tools accomplish the identification of IRs, we focused our investigation on six annotation tools (Chloë, Chloroplot, GeSeq, ORG.Annotate, PGA, and Plann) and collected the relevant articles and available source codes. We also scanned IRs from NCBI sequences using airpg [51], a tool that includes functions to identify and parse the IR annotations of plastid genomes regardless of their format. Detailed information and references on the annotation tools used in the analysis can be found in Table 10.

For each annotation tool, we reviewed the implementation code to determine the general strategy for identifying IRs. From the general strategy and implementation specifics, we inferred the behavior of the method as a function of a range of possible inputs, particularly with respect to the treatment of genome circularity and the presence of ambiguous characters in the input data. We also investigated how possible differences in IR regions are handled and what types of mismatches are tolerated.

### 4.3. Comparative Results Analysis

To annotate cpDNA sequences with all tools in a standardized way, we implemented a set of scripts, one for each tool, that have the same format for inputs and results. The input is a fasta or GenBank file with a sequence to be annotated. The results are four integer positions of IR junction point locations (i.e., start and end points of IRa and IRb), or none if no IRs were identified. The scripts were implemented as stand-alone executables in the project (https://github.com/CroP-BioDiv/irs_wrappers, accessed on 27 June 2022).

We processed the acquired cpDNA sequences using six annotation tools to identify the locations of IR. We analyzed the obtained results for properties related to identification problems, genome circularity, and mismatches in the detected regions.

For the sequences in the IRL dataset, we examined the number of identified and unidentified IRs and how the results corresponded to the documented IR loss. For the IR dataset, we analyzed the results for the following properties: (a) type of IR regions identified, (b) treatment of the circularity of the chloroplast genome, (c) treatment of sequences with ambiguous bases, and (d) differences between IRa and IRb. In addition, we investigated the distribution of the lengths of the identified regions.

Regarding the type of IR regions, we classified the sequences as follows: (a) the sequences in which the IR regions were identified and verification showed that the IRa and IRb were identical (identical IRs), (b) the sequences in which the IR regions were identified but differed (different IRs), and (c) the sequences in which the IR regions were not identified (no IRs).

The pipeline to perform the analysis was implemented in Python using the Biopython package [95]. The code is maintained in a public repository (https://github.com/CroP-BioDiv/zcitools; accessed on 31 December 2021). Figures were generated using the Python library Matplotlib [96].

## 5. Conclusions

Long inverted repeats are present in most chloroplast genomes. The location of IRs within the sequence is valuable information because cpDNA data have been used extensively in studies of plant phylogenetics. Bioinformatically, the problem of identifying IRs is easy to solve, as there are a variety of software programs designed to identify exact or similar long reverse-complement repeats. Working with public cpDNA genome sequences shows us that the assembled data are not always perfect and that there are situations where we are very confident that IRs exist, but two exact or similar long repeats cannot be found easily. The annotation tools analyzed (Chloë, Chloroplot, GeSeq, ORG.Annotate, PGA, Plann) use two approaches to solve the problem, using existing general search tools (ORG.Annotate, PGA, Plann) or implementing stand-alone solutions (Chloë, Chloroplot, GeSeq). The development of annotation tools that implement stand-alone solutions to this problem is evidence that researchers encountered data-imperfection problems before. In terms of results, all methods differ from each other, and for each combination of methods there are examples of sequences where one performs better than the other. This suggests that there is room for improvement in the identification of IRs and that a pool of ideas for this can be found in the implementation of existing annotation tools.

## Figures and Tables

**Table 1:** The description of IR identification strategies implemented in six chloroplast annotation tools presented in publications and derived from code analysis.

Method	Description in Publication	Code Analysis
Chloë	Does not mention IR identification.	Chloë assembles reverse-complement blocks of ~20 bp in size into long sequences allowing up to a few bases (≤10 bp) of mismatches.
Chloroplot	The publication contains a description of the main steps. The method can detect nonidentical IR regions allowing small stretches of mismatches.	Chloroplot assembles reverse-complement blocks of size 100 bp into long sequences, sorts the assembled sequences by position, and finds the longest gap between sequences. Assembled sequences downstream of the gap are merged into IRa, and sequences upstream of the gap are merged into IRb. Identified IRa and IRb are checked to ensure that they do not differ in length by more than 1000 bp. In addition, mismatches between the regions are calculated and checked to support the length differences between the regions. If the procedure fails, the same procedure is attempted with blocks of 1000 bp in length.
GeSeq	The minimum length for IR annotation is 200 bp. GeSeq only annotates the longest identical IR pair that matches within a submitted sequence.	GeSeq finds leftmost and rightmost exact matches with a size of at least 2000 bp and designates the regions between the match positions as IRa and IRb.
ORG.Annotate	-	ORG.Annotate uses repseek to find approximate long matches, scores these matches according to the results of a query of the input sequence in a database containing 45 LSC and 72 SSC regions, and considers the highest scoring matches as IRs.
PGA	The IR boundary annotation is performed with a self-blastn search. One parameter can be adjusted to determine IR boundaries: the minimum allowed IR length (default = 1000).	PGA performs self-blasts (-perc_identity 99) on a duplicated sequence to find the maximum inverted match with a length of at least 1000 bp.
Plann	Does not mention IR identification.	Plann performs self-blasts (-evalue 1 × 10^−200^) on an original sequence to find the maximum inverted match with a length between 10,000 and 50,000 bp.

**Table 2:** Identification of inverted repeats in sequences of the inverted repeat-lacking (IRL) dataset and the list of species with IRs larger than 10 kb detected using six annotation tools (and those annotated in NCBI).

Sequence Numbers	Chloë	Chloroplot	GeSeq	ORG.Annotate	PGA	Plann	NCBI
IRs not identified	109	96	124	4	112	130	131
	(77.30%)	(68.09%)	(87.94%)	(2.84%)	(79.43%)	(92.20%)	(92.91%)
IRs identified	32	45	17	137	29	11	10
	(22.70%)	(31.91%)	(12.06%)	(97.16%)	(20.57%)	(7.80%)	(7.09%)
IRs larger than 10 kb	11	5	10	10	10	11	4
Species (asterids/Ericaceae) with IRs larger than 10 kb							
*Agapetes malipoensis*	+	-	+	+	+	+	-
*Gaultheria griffithiana*	+	-	+	+	+	+	+
*Rhododendron delavayi*	+	-	+	+	+	+	-
*Rhododendron griersonianum*	+	-	+	+	+	+	+
*Rhododendron kawakamii*	+	-	-	+	-	+	+
*Rhododendron platypodum*	+	-	+	+	+	+	+
*Vaccinium macrocarpon*	+	+	+	+	+	+	-
*Vaccinium oldhamii*	+	+	+	+	+	+	-
Species (rosids/Geraniaceae/*Erodium*) with IRs larger than 10 kb							
*Erodium absinthoides*	+	+	+	+	+	+	-
*Erodium chrysanthum*	+	+	+	+	+	+	-
*Erodium gruinum*	+	+	+	-	+	+	-

**Table 3:** Numbers of complete chloroplast sequences classified by type of IR regions identified (identical IRs, different IRs, no IRs) using six annotation tools (and those annotated in NCBI).

	Chloë	Chloroplot	GeSeq	ORG.Annotate	PGA	Plann	NCBI
Identical IRs	3720	3558	3460	2783	3011	3148	1819
	(93.09%)	(89.04%)	(86.59%)	(69.64%)	(75.35%)	(78.78%)	(45.52%)
Different IRs	264	411	265	1212	965	795	444
	(6.61%)	(10.28%)	(6.63%)	(30.33%)	(24.15%)	(19.90%)	(11.11%)
No IRs	12	27	271	1	20	53	1733
	(0.30%)	(0.68%)	(6.78%)	(0.03%)	(0.50%)	(1.32%)	(43.37%)

**Table 4:** Number (and percentage) of complete chloroplast sequences in which the IR regions were identified using six annotation tools (and those annotated in NCBI) classified by the treatment of circularity (no wrapping vs. wrapped).

	Chloë	Chloroplot	GeSeq	ORG.Annotate	PGA	Plann	NCBI
No wrapping	3434	3386	3655	3995	3263	3943	2248
	(86.19%)	(85.31%)	(98.12%)	(100.00%)	(82.07%)	(100.00%)	(99.34%)
Wrapped	550	583	70	0	713	0	15
	(13.81%)	(14.69%)	(1.88%)		(17.93%)		(0.66%)
Total	3984	3969	3725	3995	3976	3943	2263

**Table 5:** Number of sequences in which the IR regions were identified (identical or different) using six annotation tools (and those annotated in NCBI) in the datasets of sequences with and without ambiguous characters.

	Chloë	Chloroplot	GeSeq	ORG.Annotate	PGA	Plann	NCBI
With ambiguous characters							
Identical IRs	435	375	232	325	338	363	196
	(82.70%)	(71.29%)	(44.11%)	(61.79%)	(64.26%)	(69.01%)	(37.26%)
Different IRs	86	140	67	201	180	152	72
	(16.35%)	(26.61%)	(12.74%)	(38.21%)	(34.22%)	(28.90%)	(13.39%)
No IRs	5	11	227	0	8	11	258
	(0.95%)	(2.09%)	(43.15%)	(0.00%)	(1.52%)	(2.09%)	(49.05%)
Without ambiguous characters							
Identical IRs	3285	3183	3228	2458	2673	2785	1623
	(94.67%)	(91.73%)	(93.02%)	(70.84%)	(77.03%)	(80.26%)	(46.77%)
Different IRs	178	271	198	1011	785	643	372
	(5.13%)	(7.81%)	(5.71%)	(29.13%)	(22.62%)	(15.53%)	(10.72%)
No IRs	7	16	44	1	12	42	1475
	(0.20%)	(0.46%)	(1.27%)	(0.03%)	(0.35%)	(1.21%)	(42.51%)

**Table 6:** Number of sequences with different IRs identified with six annotation tools (and those annotated in NCBI), classified by the length difference between IRa and IRb.

	Chloë	Chloroplot	GeSeq	ORG.Annotate	PGA	Plann	NCBI
IRs differ	264	411	265	1212	965	795	444
0 bp	264	177	99	816	522	377	151
	(100.00%)	(43.07%)	(37.36%)	(67.33%)	(54.09%)	(47.42%)	(34.01%)
1–10 bp	0	122	145	315	331	359	116
	(0.00%)	(29.68%)	(54.71%)	(25.99%)	(34.30%)	(45.16%)	(26.13%)
11–100 bp	0	75	17	81	111	59	117
	(0.00%)	(18.25%)	(6.42%)	(6.68%)	(11.50%)	(7.42%)	(26.35%)
>100 bp	0	37	4	0	1	0	60
	(0.00%)	(9.00%)	(1.51%)		(0.11%)		(13.51%)

**Table 7:** Number of sequences where the row method identified longer IRs regarding the column method.

	Chloë	Chloroplot	GeSeq	ORG.Annotate	PGA	Plann
Chloë		63	512	217	10	253
Chloroplot	270		573	287	61	327
GeSeq	135	12		53	13	85
ORG.Annotate	571	412	754		184	326
PGA	511	350	833	326		458
Plann	347	190	527	7	16	

**Table 8:** Complete chloroplast sequences used in the analysis.

Taxonomic Rank	Asterids (IRL/IR)	Rosids (IRL/IR)	IRL Dataset (Asterids + Rosids)	IR Dataset (Asterids + Rosids)	Total
Family	1/64	6/82	7	146	151
Genus	9/569	31/750	40	1319	1359
Species/subspecies	16/1906	125/2084	141	3990	4131
Number of sequences	16/1908	125/2088	141	3996	4137

**Table 9:** Complete chloroplast sequences included in the inverted repeat-lacking (IRL) dataset.

Clade	Family	Genus/Subclade	No. of Sequences	References
asterids	Ericaceae	9 genera ^1^	16	[77,78]
rosids	Apodanthaceae	*Pilostyles*	2	[76]
rosids	Cytinaceae	*Cytinus*	1	[79]
rosids	Fabaceae	IRLC ^2^	101	[80,81]
rosids	Geraniaceae	*Erodium*	10	[38,39]
rosids	Geraniaceae	*Monsonia*	3	[39,82,83]
rosids	Lophopyxidaceae	*Lophopyxis*	1	[84]
rosids	Putranjivaceae	2 genera ^3^	7	[84]

^1^ Genera: Agapetes, Allotropa, Gaultheria, Hemitomes, Monotropa, Monotropsis, Pityopus, Rhododendron, Vaccinium. ^2^ Dataset IRL for monophyletic Inverted repeat-lacking clade (IRLC) of the subfamily Faboideae contains data of 24 genera: *Alhagi*, *Astragalus*, *Caragana*, *Carmichaelia*, *Cicer*, *Galega*, *Glycyrrhiza*, *Halimodendron*, *Hedysarum*, *Lathyrus*, *Lens*, *Lessertia*, *Medicago*, *Melilotus*, *Onobrychis*, *Oxytropis*, *Parochetus*, *Pisum*, *Sphaerophysa*, *Tibetia*, *Trifolium*, *Trigonella*, *Vavilovia*, *Vicia*. ^3^ Genera: Drypetes, Sibangea.

**Table 10:** Chloroplast annotation tools used in the analysis.

Method	Programming Language	Last Code Change	Local	Web Application	Reference
Chloë	Julia	08.12.2020.	+	+	[66]
Chloroplot	R	19.08.2021.	+	+	[67]
GeSeq ^1^	D	18.12.2020.	-	+	[59]
ORG.Annotate	bash	08.11.2021.	+	-	-
PGA	Perl	29.10.2020.	+	-	[68]
Plann	Perl	26.01.2017.	+	-	[69]
airpg	python	17.09.2021.	+	-	[51]

^1^ For identification of IR, GeSeq uses the OGDRAW tool [92,93,94], which is part of the same CHLOROBOX set of plant-analysis tools (http://chlorobox.mpimp-golm.mpg.de, accessed on 31 December 2021).

## Data Availability

The data that support the findings of this study are openly available in GenBank at the NCBI (https://www.ncbi.nlm.nih.gov (accessed on 31 December 2021)).

## Supplementary Materials

The following supporting information can be downloaded at: https://www.mdpi.com/article/10.3390/ijms231810804/s1.
